# Supraglottic adenoid cystic carcinoma mimicking laryngeal amyloidosis: A case report

**DOI:** 10.3892/ol.2014.1984

**Published:** 2014-03-20

**Authors:** XIAOYUN QIAN, HAN ZHOU, YAJUN GU, YIFEN ZHANG, XIA GAO

**Affiliations:** 1 Department of Otorhinolaryngology, Nanjing Drum Tower Hospital, Nanjing, Jiangsu 210008, P.R. China; 2 Department of Pathology, Nanjing Drum Tower Hospital, Nanjing, Jiangsu 210008, P.R. China

**Keywords:** supraglottic adenoid cystic carcinoma, laryngeal amyloidosis

## Abstract

Supraglottic adenoid cystic carcinoma (ACC) is extremely rare and may be misdiagnosed as laryngeal amyloidosis. The present report describes a case of supraglottic ACC, which went unrecognized until histopathological examination of the neoplasm 18 months after the first presentation. The present patient presented with progressive hoarseness for half a year and initially required partial resection. Following quick regional recurrence, the patient received a total laryngectomy while refusing radiotherapy. Adjuvant post-operational traditional Chinese medicine was accepted. Over 3 years’ follow-up, there was no evidence of regional relapse or distant metastases. The present case is compared with a second case of supraglottic submucosal mass in which the signs, symptoms and examinations were similar to the first case, but that was diagnosed as laryngeal amyloidosis. Attention should be paid to submucosal masses in the larynx to prevent underlying malignancy and subsequent disease progression. Immunocytochemistry, such as p63 staining, is mandatory for making an early differential diagnosis of supraglottic ACC. Traditional Chinese medicine may be a useful adjuvant therapy for this rare disease.

## Introduction

Adenoid cystic carcinoma (ACC) of the larynx is an extremely rare disease that arises from the laryngeal glandular elements (1). Approximately two thirds of laryngeal ACCs arise in the subglottis. Compared with subglottic and glottic ACCs, supraglottic ACC is less common (2). The clinical characteristics of supraglottic ACC can be confused with those of laryngeal amyloidosis, which is a rare benign tumor. Amyloidosis refers to a variety of conditions wherein normally soluble proteins become insoluble and are deposited in the extracellular space. Amyloidosis with larynx-only localization is rare. The objective of this study is to show that the duration and progression of laryngeal ACCs may be quite variable, occasionally appearing as benign as amyloidosis, and the predilection for unnoticeable spreading may account for the locally advanced tumors and would also account for the high incidence of positive or even ‘negative’ margins on surgical specimens. Written informed consent was obtained from the patient.

## Case report

A 44-year-old woman with a six-month history of progressive hoarseness and an abnormal sensation in the throat was referred to the Nanjing Drum Tower Hospital (Nanjing, China). The patient had no history of smoking, coughing, dyspnea or dysphagia; however, it is worth noting that the patient was exposed to iron ore and coal dust for several years in the work place. In addition, the patient had a history of over five years of second-hand smoke inhalation.

**Figure 1:**
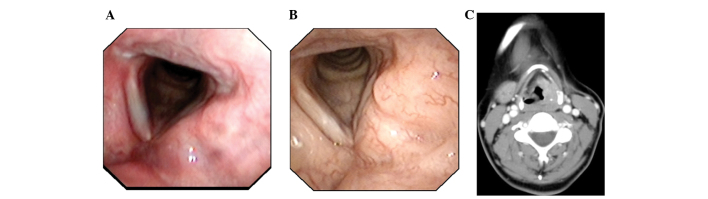
(A) Fiber optic laryngoscopy images identifying chronic congestion of the bilateral vocal folds and swelling of the left ventricular fold. The mucosa of the bilateral pyriform fossa was soft and no neoplasm was observed (18 months prior to the first surgery). (B) No obvious change one week prior to the first surgery. (C) Computed tomography of the larynx showing asymmetry of the bilateral posterior structure, an enlarged laryngeal wall beside the left pyriform fossa and a soft-tissue mass with obscure margins involving the left ventricular fold, the anterior commissure and extending to the pyriform fossa.

Fiberoptic laryngoscopy revealed swelling of the left ventricular fold and laryngeal ventricle that extended backwards to the left aryepiglottic fold. The bilateral vocal cords appeared normal and there was no vocal cord paralysis (Fig. 1A). The patient subsequently refused any further examinations. The laryngeal abnormal sensation gradually progressed, and after 18 months the patient underwent a second laryngoscopy, which revealed similar findings to the first without obvious progression (Fig. 1B). Computed tomography (CT) revealed a mass involving the left ventricular fold and anterior commissure. The left pyriform sinus was not distinguished (Fig. 1C). Neither physical examination or neck CT detected any tumescent lymph nodes in the neck, and the chest X-ray was normal.

**Figure 2:**
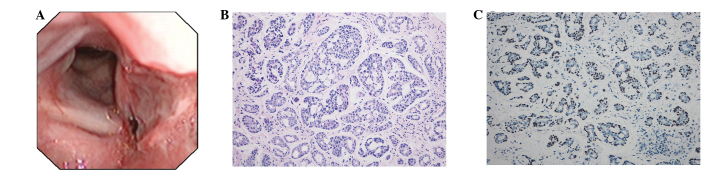
(A) Fiber optic laryngoscopy at two days post-surgery. (B) Median power view showing the mixed tubular and cribriform pattern of ACC (original magnification, ×20). (C) Expression of p63 in ACC (original magnification, ×20). ACC, adenoid cystic carcinoma.

The preoperative diagnosis that was deemed most likely was that of a laryngeal neoplasm with laryngeal amyloidosis. The subsequent frozen section biopsy of the lesion taken during laryngofissure revealed the nature of the neoplasm. As the patient refused to undergo an expanded total laryngectomy, which is indicated for a locally advanced laryngeal tumor (3), local resection of the left ventricular fold, laryngeal ventricle and aryepiglottic fold was performed only (Fig. 2A). The margin of the biopsied tissue surrounding the lesion was negative. Although there was no sign of lymphatic spreading, perineural invasion existed, therefore, it was suggested that radiotherapy be performed at one month post-surgery (4). However, one month later and prior to this radiotherapy, regional recurrence of the tumor was identified. With informed consent from the patient, a total laryngectomy without neck dissection was performed, as no lymph nodes were found to be involved (5). The tumor invaded the left side of the throat up to the root of the epiglottis, down from just beneath the true vocal cords into the subglottis ~1 cm and crossed to the right supraglottic area. The post-operative pathological findings confirmed the diagnosis of ACC and the histopathological pattern was a mixed cribriform and tubular subtype (Grade I; Fig. 2B) (6). Immunocytochemistry revealed positive staining for p63 (Fig. 2C), carcinoembryonic antigen, cytokeratin 5/6 and 8/18, epithelial membrane antigen, S100 and cytokeratin smooth muscle actin. Although adjuvant radiotherapy is known to provide superior disease control, p63 staining was positive (7,8). The patient rejected radiotherapy as it would not cure the disease, but selected traditional Chinese medicine as an adjuvant therapy. During the 42-month follow-up period, there was no evidence of regional relapse or distant metastases.

## Discussion

ACC of the head and neck typically arises in the major and minor salivary glands (9). As accessory salivary glands are exiguous in the larynx, laryngeal ACC is a rare disease, accounting for <1% of all malignant tumors in this area (10). When ACC occurs in the supraglottis, it often involves the false cords, the aryepiglottic folds and the caudal aspect of the epiglottis. In the glottis, ACCs are located in the floor of the laryngeal ventricle and subglottic surface of the anterior commissure (11). As ACCs spread in an unnoticeable submucosal and perineural fashion, early diagnosis is difficult, therefore, when a patient is referred to hospital, the disease is often at the advanced stage. In the present study, the laryngeal ACC was at stage II (T2N0M0) prior to biopsy, but at the revision surgery the tumor was at stage IV.

Grade I ACC is commonly associated with early recurrence and an earlier risk of distant metastases (12). In the present case, the extraordinary short time of recurrence may result from the misleading ‘negative’ margins, and it is possible that unrecognized invasion of specimens existed in this case. Furthermore, it may be due to the lack of adjuvant radiotherapy, although this has not yet been confirmed by randomized clinical studies. In addition, staining was positive for p63, whose alteration was an independent prognostic marker and associated with radiotherapy resistance.

Laryngeal ACC may be easily misdiagnosed as laryngeal amyloidosis, whose manifestation is similar (7). Laryngeal amyloidosis is one of the rare benign tumors of the larynx, accounting for <1% of all benign tumors of this region. Laryngeal amyloidosis is characterized by the presence of extracellular fibrillar proteins in the laryngeal tissues and may be localized without systematic manifestations. The accumulation of amyloid deposits affects the normal structure and function of laryngeal tissues (13).

In the present case, the patient was misdiagnosed with laryngeal amyloidosis due to the following: i) The medical history of the patient revealed a female non-smoker with a two-year slow progression of symptoms; ii) the symptoms included hoarseness and an uncomfortable laryngeal sensation, but not dyspnea, dysphagia or stridor; and iii) the diagnostic tests revealed normal chest X-ray results and submucosal masses involving the left ventricular fold, aryepiglottic fold and pyriform sinus without vocal cord fixation or paralysis upon fiberoptic laryngoscopy and CT. Therefore, no clear malignancy was indicated.

**Figure 3:**
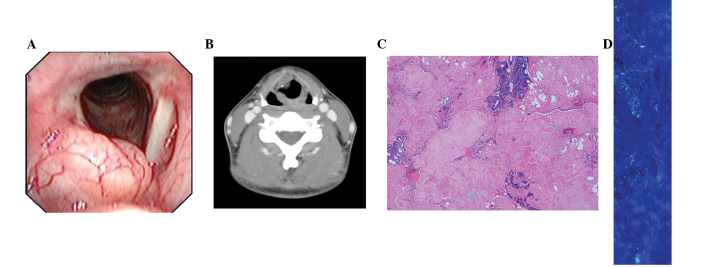
(A) Fiber optic laryngoscopy of laryngeal amyloidosis. (B) Computed tomography (CT) showing a mass in the right supraglottic region. (C) Paraffin-embedded, hematoxylin and eosin-stained tissue section showing extracellular, eosinophilic and amorphous amyloid (original magnification, ×40). (D) Paraffin-embedded, hematoxylin and eosin-stained tissue section under polarized light, with apple-green birefringence (original magnification, ×40).

In comparison, one month prior to the present case, a 33-year-old female attended an appointment at the Nanjing Drum Tower Hospital and was diagnosed with laryngeal amyloidosis. The patient presented with an abnormal sensation in the throat. The findings of the laryngoscopy and CT scan are shown in Fig. 3A and B and are similar to those of laryngeal ACC. By specific Congo red staining, amyloid appeared as a diffuse subepithelial extracellular deposit of amorphous eosinophilic material and as hyaline rings. Dense amyloid cracks in tissue sections left cleft-like spaces (Fig. 3C). Under polarized light, amyloid displayed typical apple-green birefringence (Fig. 3D).

Laryngeal ACC recurs quickly and requires sufficient management during the first treatment modality. Thus, we suggest a biopsy with specific immunocytochemical staining as the first treatment strategy. With developing molecular biology, novel therapeutic regimens may be identified from traditional Chinese medicine.

Supraglottic ACC of the larynx is a rare entity and is often disregarded. This diagnosis should be considered in patients presenting with progressive hoarseness and an abnormal sensation in the throat, even if there are no other presenting symptoms and with/without a negative chest X-ray. Pre-operative histopathological analysis is essential for an earlier differential diagnosis of laryngeal amyloidosis. In our opinion, prospective randomized multicenter studies are required to determine an optimal treatment. Traditional Chinese medicine may be a post-operative treatment regimen instead of radiotherapy; however, it requires further investigation.
